# Salicylic Acid

**Published:** 1875-06

**Authors:** W. Taft


### ARTICLE VIII.

### *Salicylic Acid.*

BY WM. TAFT.

Among the many new remedies and the many new applications of old ones that are being constantly introduced into medicine and dentistry, few bid fair to become more prominent than salicylic acid as an antiseptic and disinfectant. Prof. Kolbe, to whom the credit of having introduced it into practice as an antiseptic belongs, had his attention called to it from the fact that it could be readily prepared from carbolic and carbonic acid, and supposed that like them it would retard or entirely prevent fermenting and putrefactive decompositions. He instituted a series of experiments the results of which confirmed his supposition to a surprising degree. He introduced it into the Lying-in Hospital at Leipsic, where it was used with good success. In an article written under the title of salicylic acid, and for the *Deutsche Vierteljahrsschrift zur Zahnheilkunde*, by Dr. Osterman, of Brunswick, reference is made to the experiments of Prof. Kolbe, and says that in consequence of the successful results of the use of salicylic acid in the hospital, he was led to investigate it in its application to dentistry. Dr. Osterman first communicated the results of his observations at the annual meeting of the Central Society of German Dentists.

The following extract translated from the article alluded to, contains some of Dr. O's observations concerning the application of salicylic acid to dentistry.

"In cases where the pulps of the teeth are changed through suppuration and gangrene into a foul, disagreeably smelling, gas evolving mass, it is well known that if they are filled without first restoring them to a healthy condition, periostitis will in all probability result. In such cases I introduce into the nerve canal dry salicylic acid and then make a temporary filling which I allow to remain for several days. In order to make the stopping more secure, I saturate a piece of spunk with an ethereal solution of salicylic acid and with it fill the pulp chamber. The results in a large number of cases were good. The decaying, offensive nerves in the root canals become fully deodorised and shrink into a mumified detritus. Such detrital matter can neither by evolution of gas or putrid secretions irritate through the apicial foramen. I will here remark that it may be difficult to effectively introduce the dry salicylic acid into the root canals. If such be the case we can have recourse with advantage to a concentrated solution of the acid in ether, which will volatilize in a very few moments. This course will be especially indicated in the molar teeth; in the incisor teeth and pulp cavities where cleansing can be conveniently performed it should never be neglected. After washing the canals with the above solution, we saturate a piece of spunk, place in the bottom, and then fill with any material that may be desirable.

In the treatment of suppurating pulps, dry salicylic acid may be employed with advantage. In erosion and in inflamed conditions of the mucous membrane and jaws, I have applied salicylic acid with success. In stomacacea and scorbutic inflammation of the jaws, where the borders and inter-dental papillæ appear degenerated, gangrenous, and are coated with a putrid stringy secretion, I have applied salicylic acid mixed with equal parts of the powdered cassia and cinnamonum with a soft brush. The foul taste accompanying such conditions, also the ill smelling breath soon disappear. It may here be added that in every inflammatory condition caused by roots dead or affected by periostitis salicylic acid will prove an admirable remedy.

Aside from the application of salicylic acid in special cases, I have employed it as an every day means of purifying the teeth and mouth, with the best results.

For a disinfecting mouth-wash: One part of salicylic acid to three hundred parts of water will be sufficient, or if a stronger solution is required, it may be prepared by adding three parts of phosphate of soda and thirty parts of distilled water to one of salicylic acid. It forms a valuable addition to most tinctures and tooth powders.—*Dental Register.*

---

